# Case of Hourglass Contraction before Delivery

**Published:** 1866-04

**Authors:** D. L. Crist

**Affiliations:** Bloomington, Ill.


*Case of Hourglass Contraction before Delivery.* By D. L. Crist, M. D., Bloomington, Ill. (Reported by C. Jud. Gill, M. D.)

Mrs. McG——, aged 45 years, the mother of six children, was taken with labor pains early on the morning of Feb. 15th. Saw her at 9 o'clock in the evening. On examination found the os dilated to the size of a silver dollar, and in good condition. Pains regular and strong. At 10 o'clock the membranes ruptured, and an unusual amount (I should think not less than a gallon) of liquor amnii gushed out. An examination was now made and a breech presentation recognized.

At the next pain another gush of water, not so violent or copious as the former, but yet quite free, took place. The case was now left to nature about an hour, the pains continuing regular and powerful. Another examination was then made and the os found well dilated, but no progress in the labor otherwise. At each succeeding pain the head came down into the true pelvis, but immediately retracted above the superior strait as soon as the pain ceased. Desiring to ascertain, if possible, the cause of delay, I passed the left hand into the uterus, along the abdomen of the child, until I reached about the point of the ensiform cartilage, where I found the body was firmly grasped by a contraction in the uterine walls. The feet were above and firmly held by the stricture, while the umbilicus was below. With some difficulty I succeeded in passing the finger through the stricture between the child's feet, and attempted to overcome the contraction by continued pressure, but failed. After giving the patient a rest for half an hour, a tape was passed around the thighs of the child, and, not being provided with chloroform, I administered antimony to the extent of producing emesis. Steady traction was then made for about half an hour, when the feet came down suddenly, and very soon with about half the body appeared externally. The progress of the labor here became arrested and remained so nearly an hour. The pains being weak and ineffectual I administered ergot, and as soon as they became strong made vigorous traction and soon succeeded in effecting delivery. The child was dead. The placenta was delivered without difficulty. The patient recovered with no unusual symptoms.

If this were a case of hourglass contraction, what was the probable cause? I can think of none, unless it be the unusual amount and sudden expulsion of the liquor amnii.

